# The Phoenix Heart—PICSO and the Rebirth of Embryonic Life in the Ischemic Myocardium

**DOI:** 10.3390/jcdd13020060

**Published:** 2026-01-23

**Authors:** Werner Mohl, Leonie Fanny Steingruber, Dejan Milasinovic, Angela Simeone, Vilas Wagh

**Affiliations:** 1 Department of Cardiac Surgery, Medical University of Vienna, 1090 Vienna, Austria; 2 WMohlMedConsult Lab, Lazarettgasse 12/19, 1090 Vienna, Austria; 3 Faculty of Medicine, University of Belgrade, University Clinical Center of Serbia, Visegradska 26, 11000 Belgrade, Serbia; 4 Qiagen Ltd., Manchester M13 0BH, UK; 5 Merck Research Labs, Boston, MA 02115, USA

**Keywords:** embryonic recall hypothesis, PICSO, myocardial ischemia and infarction, infarct size reduction, cardiac regeneration, heart failure therapies, circulating noncoding RNA

## Abstract

Pressure-controlled intermittent coronary sinus occlusion (PICSO) was initially developed to salvage ischemic myocardium. However, recent evidence suggests a more profound role: reawakening embryonic molecular pathways that facilitate myocardial regeneration. This review examines the paradigm shift in PICSO’s mechanism—from its traditional focus on infarct size reduction to its emerging role as a catalyst for myocardial repair through the reactivation of embryonic signaling. Findings suggested that myocardial decay could be ameliorated beyond salvage, revealing that PICSO enhances vascular activation in the coronary venous system, thereby influencing the fate of endothelial and myocardial cells. The theorem “embryonic recall” posits that PICSO induces molecular signals reminiscent of early cardiac development, offering a novel approach to cardiac repair in myocardial jeopardy. Noncoding RNA serves as a universal signaling event, thereby supporting the hypothesis. Yet, conflicting clinical outcomes highlight the need to redefine PICSO’s objectives, optimize device settings, and realize interventional strategies. The evolution of PICSO demands a radical shift in scientific perspective. Beyond ischemic salvage, its true potential may lie in harnessing regenerative mechanisms within the failing heart. Modern cardiology must adopt this dual role, bridging mechanical intervention with molecular rejuvenation to ensure its continued viability as a therapeutic option. PICSO, like the phoenix, may yet rise anew as a transformative force in cardiovascular medicine.

## 1. Introduction

“Time is muscle,” as extensively proven in many clinical studies, is the basis of state-of-the-art therapy for myocardial infarction today [1,2]. Reperfusion injury, as a counterintuitive consequence of myocardial jeopardy, was identified, and numerous studies have sought to reduce this threat [3]. Despite many trials, concepts to further reduce infarct size or regenerate the failing heart have failed to translate to clinical practice.

Restoring myocardial function in the setting of myocardial jeopardy by inducing structural regeneration has been the dream of scientists for decades. Ever since cellular therapies showed promise of myocardial regeneration, a multitude of concepts, formulas, and therapeutic avenues have been tried with variable success. A novel approach may be to mimic cardiac development in adult failing hearts. Morphogenesis in mammalian hearts is a stepwise signaling matrix that unfolds genetic information into the form and function of cardiac structures. Controlling the spatiotemporal diversification of the genome into normal or congenital aberrant phenotypes involves feedback from flow patterns in developing hearts. Thus, using this feedback loop to reverse hemodynamic stimuli into molecular signals via activation of venous vasculature may facilitate re-entry of morphogenetic processes in adult failing hearts.

In this article, we summarize the available knowledge supporting this concept, which is based on our previously published theorem, “Embryonic Recall” integrating in the context of other concepts claiming cardiac regeneration [4].

Considering the logistical limitations of cell transplantation, a comprehensive molecular signaling approach via periodic reversal of venous flow in the cardiac microcirculation, using trans-coronary sinus interventions, may become a practical alternative to induce myocardial regeneration.

None of the pharmacological or device studies gained widespread clinical acceptance, leaving timely reperfusion of ischemic myocardium as the only treatment choice. There is, however, an unmet clinical need, given the remaining obstructed microcirculation that hinders nutritive flow to the reperfused myocardium [5,6,7]. This leads to a preventable increase in debilitating symptoms of heart failure and even death.

End-stage heart failure therapy is another critical clinical need, and several trials to regenerate the heart have fallen short of delivering optimal recovery. Today’s emphasis is mainly on logistics and digital remote monitoring, or, as a last resort, left ventricular support or heart transplantation. Besides salvaging the ischemic myocardium and reducing the negative consequences of myocardial decay, it is also essential to know how to provide timely therapy rather than to search for new ways to regenerate the heart. The promise that the heart may regenerate with cellular therapy never reached momentum. Although cellular therapy to regenerate the heart never reached widespread application, the impasse created hostility for further research [8]. There are still questions about whether this constitutes a conceptual failure or a loss in transition; heart failure therapy still suffers from a gap between medical treatment and supporting devices [9,10]. In 2011, Pereira, in an article on exploiting options for regeneration after myocardial infarction, defined a time frame ranging from hours to months, depending on the target for repair [11]. Therefore, timely intervention shortly after myocardial injury, which extends the window of opportunity for effective repair, remains an unmet clinical need. Although cardiac surgeons who arrest the heart during procedures have learned that managing reperfusion and controlling the reperfusate can reduce reperfusion injury, this remains an unmet clinical need. Modifying reperfusate temperature, ionic composition—particularly limiting extracellular calcium—and favoring blood-based, substrate-enriched solutions with controlled low-pressure flow can attenuate myocardial ischemia–reperfusion injury by reducing mitochondrial calcium overload, oxidative stress, and endothelial dysfunction [12]. In contrast, leaving the heart unprotected causes irreversible damage. It appears that the common denominators in the clinical understanding of reperfusion injury are microvascular obstruction and myocardial resistance [7]. A treatment combining reversal of early changes during reperfusion and reversal of cardiac jeopardy in the chronic state, towards eventual heart regeneration, is warranted. However, we also need to consider potential therapeutic targets to reverse the damage and support myocardial healing. Given the ambivalent results of cardiac regeneration from early reperfusion to regeneration efforts, an intervention like PICSO (pressure-controlled intermittent coronary sinus occlusion), known to clear the ischemic microcirculation, one of the focus areas of the management of STEMI, and to induce regenerative impulses in chronic heart failure patients, is a stimulating alternative to more complex treatment concepts [5,13].

## 2. PICSO in the Context of Cardiac Jeopardy

PICSO, one of the potential candidates for improving microcirculation, underwent a significant paradigm change early in the translation of extensive scientific evidence on myocardial salvage into a better understanding of the underlying molecular mechanisms and their potential to reduce the burden of myocardial decay and promote regeneration. In the early days of PICSO research, myocardial salvage was the holy grail; however, concomitant success in interventional reperfusion shifted the focus. Additionally, defining the salvage potential of PICSO clinically became problematic.

It is widely believed that in acute myocardial infarction, patients with microvascular obstruction experience subsequent increases in myocardial resistance, which in turn affect patient outcomes. Today, two critical issues remain unresolved. Restoring the reperfused microcirculation not only speeds infarct healing but also promotes self-healing pathways that can regenerate the heart.

Although the knowledge that PICSO can treat intramyocardial resistance has been known for decades, the pathophysiology of the reperfused microcirculation was previously neglected, as was the notion that most of these changes primarily affect cardiac veins [14,15].

The concept of PICSO was first formulated during the early days of thrombolysis and early revascularization [16]. In these early days, infarct size reduction was demonstrated experimentally using the original planimetric area-at-risk and infarct size determination in canine experiments. It reached 45% in a highly significant infarct size reduction when treatment started 15 min after experimental induction of ischemia. The following experimental results demonstrated a decrease in infarct size and improved regional function when the application was initiated early during ischemia [17]. A meta-analysis documented a thirty percent reduction in infarct size across different species with early onset of therapy in experimental infarction [18]. Applying PICSO during reperfusion has been studied in only one early clinical study during bypass surgery and in a pilot canine study with experimental infarction [19,20].

Another clinical study is also worth mentioning in this context. PICSO, in a manually controlled form of intermittent coronary sinus occlusion named ICSO, applied during lytic therapy, was found to limit infarct size. Two critical differences exist between today’s state-of-the-art treatment for acute coronary syndromes and the protocol in this study. First, the study employed different infarct size measurements. Second, PICSO, in its manually controlled form without automatic pressure control, was applied during ischemia in a trial involving lytic therapy [21]. When combined with lytic therapy, the time to reperfusion was significantly shorter than in the control group (*p* = 0.014). Long-term data showed significant differences in reinfarction (*p* = 0.015) and major adverse cardiovascular events (*p* < 0.0001) between the two groups, exceeding all expectations, therefore unrelated to the amount of salvage.

This finding was counterintuitive to the earlier scientific understanding and necessitated a thorough examination of the PICSO concept.

**Figure 1:**
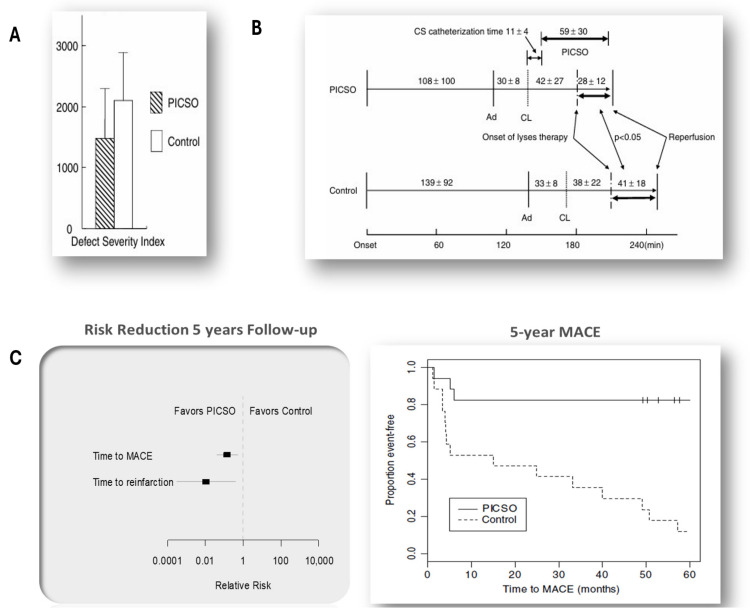
The long-term evaluation was a key point in how PICSO was viewed. The data analysis showed that patients benefited from additional PICSO effects beyond traditional salvage. Panels (**A**,**B**) depict salvage using Thallium defects and (**B**) shows the reduced time until reperfusion, (**C**) the analysis of risk reduction after 5 years of follow-up, as well as the reduction in MACE [21,22].

The findings regarding the marked reduction in major adverse cardiovascular events (MACE) five years after the application of PICSO in patients with acute myocardial infarction are surprising. They suggest a secondary mechanism of action beyond simple salvage, challenging the traditional view that only two “mechanistic” actions are at play. This indicates a need for a broader perspective that includes molecular actions, retrograde access to the ischemic circulation, redistribution of blood flow, and clearance of the reperfused microcirculation, thus adding confirmatory clinical data to the described hypothesis of the acute effects of PICSO on coronary microcirculation, but were also the first indication of a long-term regenerative signal [22]. Summarized results are depicted in Figure 1. The reduction in MACE and restenosis in the era before PPCI illustrates the natural course of the post-MI healing process and seems to be related to a decrease in inflammation during the healing course of myocardial infarction [23,24]. Imbesi and Berkhoff found that persistent thromboinflammation is associated with clinical outcome [23,24]. PICSO claims to interfere with the pro-inflammatory process. Pivoting from proinflammatory to advanced healing seems to be involved in analyzing results on MACE and restenosis.

Searching for the best therapies in myocardial jeopardy involves turning experimental medicine into clinical practice. However, many reasons exist why a promising idea fails in clinical trials or as a routine treatment. Recently, another randomized, well-designed clinical trial aimed at reducing reperfusion injury and infarct size was unable to provide convincing evidence supporting PICSO as an addition to primary PCI in everyday practice [25]. This early-stopped trial, conducted during the COVID-19 pandemic, showed no difference in infarct size at three days or in follow-up. Several factors influence the outcome, as evident in the subgroup analysis of pre- and post-COVID patients; however, two variables are most important. First, differences in how experimental and clinical infarct sizes are measured, along with the known effects of PICSO on perfusion area and area at risk, need to be considered [19,25]. Second, the timing of the PICSO application after reperfusion is crucial. In the initial clinical surgical setting trial, PICSO was initiated 5 min after global ischemic arrest during proximal bypass anastomoses [20]. Documented improvements in regional function can be explained by PICSO effects, redistributing flow towards limited perfusion zones before bypass opening. All other studies were conducted during the early stages of experimental coronary artery occlusion. Only one study on PICSO has been published during reperfusion in an experimental canine experiment, documenting substantial salvage [19].

## 3. The Embryonic Recall “Theorem” Decoding Cardiac Structural Regeneration

By revising Braunwald’s established principles [1], the PICSO concept demonstrates three significant effects that help reverse myocardial decline. First, it redirects retrograde blood flow into areas of the myocardium that are deprived of adequate circulation, clears blocked microcirculatory channels, and activates key molecular signals [26,27].

Decoding cardiac structural regeneration requires accounting for the process of healing [28]. There are many different counteracting processes involved, but after several months, the infarct size in reperfused myocardial infarctions decreases substantially.

Bertero described the most important hallmarks needed for clinical significance, to be neo-angiogenesis, remuscularization, immunomodulation, electromechanical stabilization, and resolution of fibrosis [29]. To date, regenerative efforts shift from cellular involvement, such as cellular injections or derived signals, towards secreted molecules [30]. Soczyńska [31] concluded in a recently published paper and summarized that: “*Clinical data concerning therapies based on cellular signals, while sometimes inconclusive, often yield outcomes comparable to or even superior to those of cell-based interventions. Nonetheless, both approaches face substantial challenges, including ensuring the reproducibility of results, standardizing therapeutic product preparation, and addressing ethical and regulatory considerations.*” Considering the new options, shifting evidence from cellular to “in vivo reprogramming and extracellular vesicles,” we believe the current notion of including secreted molecules in regenerative therapy is promising and aligns with our conceptual background [31].

Endogenous repair involves reviving dormant repair mechanisms in the adult failing heart, primarily by prolonging or reactivating natural signaling pathways. It remains an open question why mammalian hearts, unlike more primitive reptilian hearts, lose the ability to regenerate adequately. Undeniably, the complex structure of mammalian hearts and their functional homeostasis do not allow cellular replacement on a large, clinically scalable scale, although limited turnover persists throughout life [32]. Hence, the “paracrine effect” superseded earlier theories, including the recruitment of cardiac stem cells from niches rather than direct myocardial regeneration, which represents endogenous repair. Paracrine effects remain a matter of debate, and current theory includes a plethora of molecular signals, including exosomes, small molecules, and specific non-coding RNAs and miRNAs [33,34]. Yaniv’s [33] core concept reiterates Monod’s operon model, in which genes for related functions are grouped, controlled by repressors binding to operator sites, and induced by small molecules [35]. Other avenues for research into inducing endogenous repair followed [36].

PICSO and the embryonic recall theorem claim that several regenerative processes exhibit irreducible complexity in the technology used, meaning that all components must work together to achieve the desired outcome. Knowledge of the basis of this procedure necessitated alternative thinking and predicted drastic changes. The activation of the endothelium in cardiac veins and the subsequent molecular signaling were anticipated [26]. The search and ideation for adequate molecular signals and pathways started. We predicted that these signals are of embryonic origin and have been conserved throughout mammalian evolution. Evidence was published that non-coding RNA and other transcription factors are plausible candidates [37,38]. Our hypothesis, “embryonic recall,” suggests that regenerative impulses reproduce the embryonic effects that drive cardiac development, particularly muscularization and angiogenesis, which are lost in the failing heart. We propose that mechanotransduction and mechanochemical processes are involved [4,39]. Mechanotransduction of flowing blood is known as the “force within” and as the input signal that shapes the developing heart [40].

Spatiotemporal shear stress patterns on the primitive endocardium are involved in major stages of cardiac development, from the onset of the heartbeat, coinciding with looping and therefore with the onset of blood flow. Shear stress from flowing blood, sensed by primary cilia and transmitted via the cytoskeleton of endothelial cells into molecular pathways, is a key epigenetic factor in cell biology [41].

We theorize that mechanosensation in the developing endocardium closes the feedback loop between establishing blood flow by the primitive heart tube and gene regulatory networks via molecular signaling, as shown by Miyasaka [42]. This “developmental episode” is the authoritative dualistic command that connects the genome, environment, and structured staging, needed to stabilize evolutionary concealed body plans.

Activation of an embryonic recall program was observed in cardiomyocytes, characterized by the re-engagement of mechanochemical, epigenetic signaling processes typically restricted to early development. Mechanical cues, including substrate stiffness and cyclical stretch, along with altered calcium handling and actin–myosin remodeling, triggered the reactivation of embryonic gene networks linked to proliferative and plastic states. This change was accompanied by increased metabolic flexibility and a partial reversal of terminal differentiation, showing that mechanochemical signaling can mimic developmental programs to enhance adaptation and regenerative potential in mature cardiomyocytes [43,44]. We propose that scientific evidence for mechanisms identified during organogenesis supports our theory of PICSO in the adult failing heart [41]. Moreover, Miyasaki found that miRNA 143 expression was dependent on the heartbeat, meaning that the evolving function was necessary to close the mechanochemical circuit.

**Figure 2:**
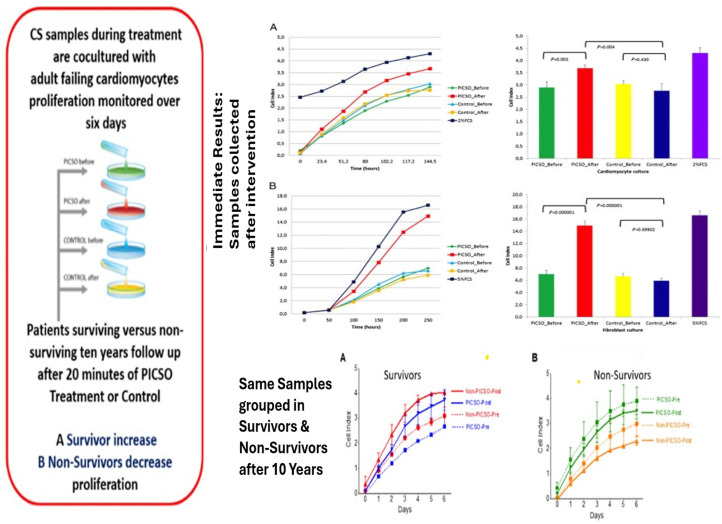
Schematic of the PICSO HF trial [45,46]. Blood samples were collected from the coronary sinus before and after the PICSO intervention, or from the equivalent 20 min period in controls. These sera were cocultured with myocardium sampled from heart failure patients during LVAD implantation from the apex of the heart. Comparing samples before and after intervention, PICSO and non-PICSO, as well as for survivors and non-survivors, showed distinct differences in behavior, suggesting proliferation in survivors and PICSO patients. The left panel outlines the methodology used to demonstrate that the cell index serves as a surrogate for the PICSO intervention’s proliferation potential. The upper right shows the immediate effects on the cell index, with grouped statistics on the right. The lower part shows regrouping samples by patient survival data. Real-time cell index for (**A**) human primary cardiomyocytes sampled from an explanted heart from a patient with heart failure during transplantation and (**B**) commercially available fibroblasts cultured in the presence of PICSO (n = 5 patients) or non-PICSO (n = 6 patients) sera from pre-procedure and post-procedure. Cell index was measured for up to 250 h for fibroblasts and for up to 144 h for primary cardiomyocytes. Student’s *t*-test significance is shown in the graphs on the right for comparisons among groups at the end of the measurement periods. Note that post-PICSO sera induced additional proliferation, whereas post-control sera showed no change. Regrouping the same samples by survival reveals an interesting prognostic relationship between patients who survived and those who did not at the time of the intervention. Note the difference between before and after the intervention, or between the respective time periods, in controls. Modified from [45,46].

In analogy, we found a similar pattern of non-coding RNA expression and transcription factor expression in a clinical study of heart failure patients with and without 20 min of PICSO [45]. Investigating the ten-year outcome of these patients found that four miRNAs involved in cell cycle, proliferation, morphogenesis, embryonic development, and apoptosis were significantly increased in survivors and PICSO, whereas they decreased in non-survivors. In contrast, three miRNAs involved in proliferation and survival, cell-fate determination, and endosomal recycling decreased in survivors and PICSO. In vitro cellular proliferation increased in survivors and decreased in non-survivors, exhibiting a distinct survival pattern that discriminated against patients with up to 10-year survival in heart failure [46]. The results of this first-in-human trial on PICSO in heart failure are depicted in Figure 2.

The findings mentioned above represent a paradigm shift from PICSO effects, which reduce infarct size, to the first evidence of signals that induce myocardial regeneration. [13,45].

## 4. The Clinical Relevance of PICSO in Heart Failure and ACS?

After several decades of PICSO research, the pivotal study on this intervention failed to meet the expected endpoints. The study by De Maria certainly is a blow to the PICSO application in ACS patients, but is the evidence it provides at the end of decades of research [25,47]? Perhaps challenging the therapeutic dogma of an intention-to-treat study and meticulously following the scientific evidence of randomized studies in search of underlying confounders, in the context of previous findings, dictates a second look at the intervention per se, leading to another beginning. Reintroducing the concept of “pressure-controlled intermittent coronary sinus occlusion” to its fundamentals—such as “redistribution of flow into deprived zones and clearing of obstructed microcirculation” alongside the molecular findings will rebuild confidence and boost willingness to accept the paradigm shift and adopt the idea. In the subgroup analysis of the PiCSO I AMI trial, zero TIMI flow at presentation favored PICSO, aligning with the authors’ earlier findings in the OXAMI trial, which indicate that effective clearing of reperfused microcirculation remains a crucial therapeutic goal [7]. We should not confuse “clinical relevance” with confounders such as market perspectives and shareholder expectations, as this may obscure the true value of a procedure.

**Figure 3:**
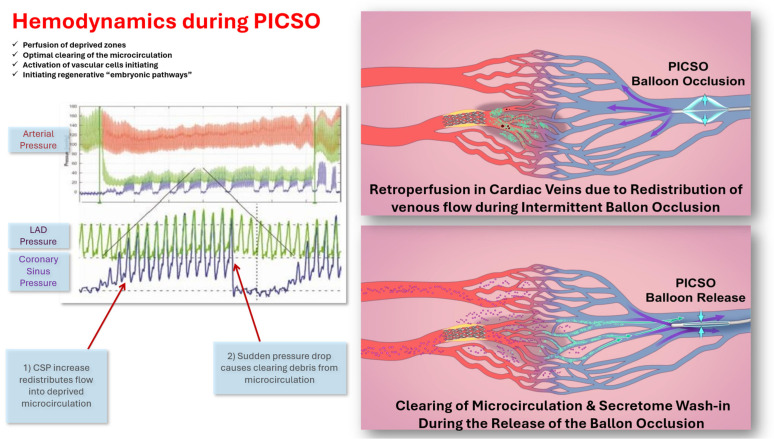
Debris from the microcirculation is cleared during the release of the coronary sinus blockade. Note the concomitant increase in post-occlusive LAD pressure and coronary sinus pressure as an indication of retrograde filling of the deprived zones. The combination of retrograde access of the deprived zone seen by the elevation in the post occlusive coronary artery pressure, the plasma skimming effect in the microcirculation, the clearing from debris, and lowering the vascular resistance acts in combination with the excretion of molecular signaling by stretching of pericytes, and bending cilia in cardiac veins according to the oscillating venous flow initiating regenerative pulses in adult failing hearts. Modified from Mohl Frontiers 2023 [13].

Without any doubt, the washout effect of PICSO is clinically relevant. First, documented in Kenner’s seminal paper and corroborated clinically by de Maria’s Oxami-PICSO study and van de Hoeft’s observation on IMR, this intervention is important for the ischemic microcirculation, as depicted in Figure 3 [7,15,48]. The general observation from this clinical observation was that IMR-guided treatment of anterior STEMI with PICSO improved microvascular function and reduced infarct size [49].

Reflecting on previous preclinical and clinical experiences and considering the differences in the concepts of measuring area at risk and infarct size clinically may have altered expectations in randomized trials. All data collected over decades of research indicate that PICSO performs best during ischemia and only slightly during reperfusion. The consequence of De Maria’s latest publication was that no adjunct therapy is needed with modern reperfusion technology [25,47]. Still, it was also an eye-opener that new technology is required to align needs, as the application in the period before interventional reperfusion may enhance the potential of PICSO in acute coronary syndromes (ACS). As soon as this new technology becomes available, new results supported by numerous previous beneficial scientific reports, both experimental and clinical, will change the landscape of PICSO in ACS.

**Figure 4:**
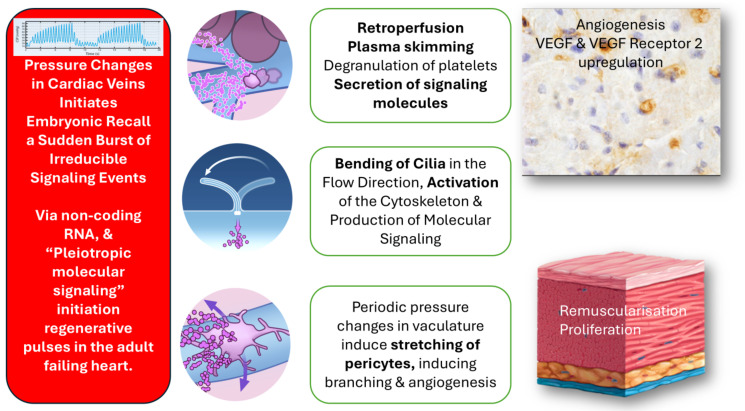
Schematic of the embryonic recall hypothesis claiming that activation of venous endothelium during PICSO induces a burst of mechanochemical signaling events reconnecting to similar embryonic pathways. Plasma skimming with degranulation of platelets and retroperfusion directly in the microcirculation of the failing heart is combined with cilia deflection of oscillating flow in cardiac veins, leading to the expression of angiogenic pathways, as evidenced by the increase in VEGF and VEGF2 receptors. The stretch in pericytes is another critical factor in the sudden rise in signaling, inducing pathways analogous to those in the early embryo.

Primary myocardial injury is a complex issue involving secondary effects on the myocardium, primarily remodeling, as well as overall circulatory impacts and neurohumoral factors. Most of the consequences are compensatory but eventually lead to structural changes that create a vicious cycle [50]. This adaptation process, which combines healing and fighting further decay, takes months to reach a steady state but often regresses into ongoing deterioration and myocardial decay. The PICSO application, based on the embryonic recall theorem, which claims to initiate regenerative pathways in adult failing hearts, has another aspect of clinical significance. Considering the numerous structural changes in the myocardium, coronary vasculature, and cardiac matrix, all the visualized PICSO effects shown in Figure 4 support the idea of reversing myocardial decay. The repeated embryonic signaling process may alter cardiac deterioration, as proposed in our heart failure cohort. Mechanotransduction of elevated hemodynamic pressure in cardiac veins, along with oscillating flow, is the driving force behind the PICSO intervention and resembles the situation during the sculpting of the embryonic heart [44]. The primary claim of PICSO in this setting is depicted in Figure 4, showing oscillating flow and cilia deflection as a significant determinant of development, as described in developing vasculature in zebrafish [51]. Plasma skimming in the venous vasculature, as described during PICSO in the adult failing heart, reinstitutes the described situation in developing zebrafish [15]. As described here, the secretion of non-coding RNA appears to be a significant factor influencing the proliferation of cardiomyocytes in failing hearts. These microRNAs are associated with signaling mechanisms that influence cardiomyocyte proliferation and survival, and their modulation by PICSO was linked to enhanced cellular proliferation in survivors, highlighting the potential impact on long-term clinical outcomes. This molecular pattern was independently validated in preclinical models, further confirming the regenerative effect induced by PICSO and the specificity of these miRNA signals [46]. As shown in experiments, PICSO also induces angiogenic factors, likely not limited to VEGF and heme oxygenase [52].

The claims of the embryonic recall hypothesis are summarized as follows:

1. Mechanisms of hemodynamic changes in cardiac veins reiterate embryonic signals.
2. These signals are produced in a rapid burst fashion within the heart and retrogradely flow into the cardiac microcirculation.
3. These signals comprise different parameters from non-coding RNA to growth and transcription factors and exosomes
4. The route towards deprived microcirculation and venules, where substance and cellular migration are possible, and the sudden increase in this transport towards the failing myocardium is the central claim of initiating embryonic pathways in the failing heart.

As depicted in Figure 4, reconnecting cardiac organogenesis and regeneration with vascular flow, a crucial driver of morphogenesis, supports our theorem and PICSO effects in failing human hearts, emphasizing their provocative and clinical significance [28].

Several of the clinically essential consequences of regenerative efforts, as described by Bertero, are validated [29]. Mayr found a reduction in arrhythmias in an experimental porcine model of PICSO [53]. Analysis of the clinical outcome of the Japanese study on PICSO suggests modulation of thomboinflammation leading to reduction in MACE and restenosis [22]. Studies from our research group, i.e., Wagh, investigated the signaling and the changes in cell count in human failing hearts, suggesting proliferation of cardiomyocytes and a potential survival benefit after 10 years, as well as an expression of signals that reduce fibrotic remodeling [13,46].

Therefore, with the caveat that further scrutiny of additional data is warranted, the currently available data lead us to propose our current hypothesis as a valuable explanation of PICSO effects validated in cardiac jeopardy.

Cardiac foetal reprogramming as an adaptation to heart failure symptoms and partial reversal of myocardial decay via the innate repair pathway are well known [54]. However, key information needed to translate these findings into therapy is missing, even though fetal isoforms, for instance, in cardiac fibrosis, are well described [55,56,57]. Findings span cardiomyocyte proliferation to cardiac metabolism, a crucial parameter in heart failure and ischemia. Collectively, decades of work demonstrate that heart failure and acute myocardial infarction elicit reactivation of embryonic and fetal programs across multiple cellular dimensions [58]. In cardiomyocytes, this includes well-described shifts in gene expression and splicing toward fetal isoforms, metabolic and mitochondrial remodeling reminiscent of the embryonic heart, epigenetic reconfiguration of developmental loci, sarcomere disassembly, and partial re-engagement of cell-cycle activity. In parallel, non-cardiomyocyte compartments undergo profound remodeling, encompassing extracellular matrix reorganization, altered mechanical signaling, angiogenic responses, and dynamic activation of fibroblasts and immune cells [57]. Together, these changes support the concept that cardiac injury does not induce a random degenerative state but instead triggers a conserved, developmentally encoded response.

The question remains: why do these adaptive mechanisms not heal and halt cardiac decay?

The embryonic recall hypothesis does not dispute these observations but reframes them as components of a conserved, developmentally encoded response that can be rapidly and synchronously re-engaged following injury. In contrast to the slow, cumulative, and often maladaptive remodeling and the obvious regenerative impulses and processes that characterize chronic disease progression, embryonic recall posits a temporally condensed burst of regenerative activity in which a massive influx of signals, produced by compact, irreducible complex mechanisms of PICSO effects, creates an environment in which canonical developmental pathways are simultaneously activated across multiple cardiac cell types. Effects on vasoactive molecules in border zones in experimental animals, as well as on cardiomyocyte plasticity, are clearly related to outcome and support our theorem. This distinguishes embryonic recall from concepts that primarily modulate hemodynamic and metabolic conditions to influence repair gradually. Rather than inducing incremental adaptations, embryonic recall proposes that coordinated activation of developmental gene regulatory networks—analogous to those operating during embryogenesis—can counteract pathological remodeling by reinstating a transient, regenerative state. Thus, embryonic recall represents a multifactorial yet synchronized regenerative framework that integrates cardiomyocyte plasticity with microenvironmental reprogramming and provides a conceptual advance beyond models of slow disease adaptation or isolated mechanotransductive interventions.

Extensive prior work has shown that cardiac injury induces reactivation of fetal and embryonic programs in cardiomyocytes and non-myocyte compartments, including transcriptional, metabolic, epigenetic, structural, and microenvironmental remodeling [58,59].

**Figure 5:**
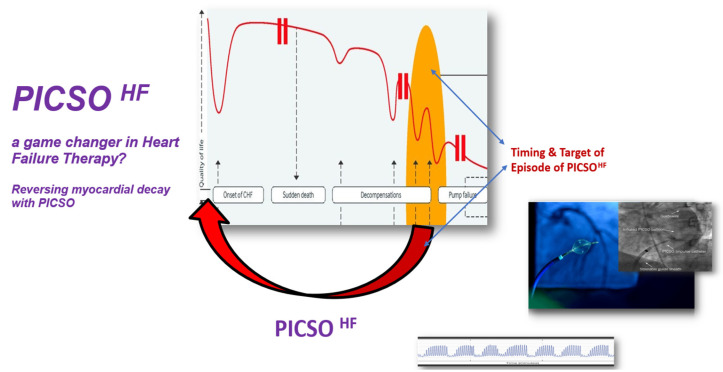
Intended use of PICSO in HF. Brief use of PICSO in this patient group might reverse the decay and improve the quality of life. Application times and the number of reversal cycles are the subject of additional research. Based on the recent findings of PICSO in HF [13,45,46], the stages of disease progression and clinical decline in patients, from disease onset to advanced therapies (i.e., first decompensation leading to the need for cardiac support and heart transplantation), are depicted. Additionally, the window of therapy is highlighted, closing the gap between medical treatment and cardiac support and reversing symptoms using PICSO.

It may realize the potential of cellular transplantation, as paracrine effects are believed to contribute to positive outcomes in a “hit and run” mode of cells, inducing epigenetic modulation [39]. This could be similar to PICSO effects and the embryonic recall theorem, which suggest that flow-induced signaling triggers repair. Its importance is vital for advancing medical research and enhancing patient care. This stimulus-triggered reconnection to embryonic pathways might serve as an alternative approach in cardiac repair. The idea that a cardiac intervention without biohazards can induce molecular signaling in failing hearts, even with a relatively short application duration, warrants initiating pivotal clinical trials. Since treating heart failure remains an unresolved therapeutic challenge, recent studies of PICSO effects indicate its potential for this indication [32]. Figure 5 illustrates the proposed use of PICSO in heart failure patients. The observational study on a 10-year follow-up of PICSO use demonstrated that all patients in the control group with ischemic cardiomyopathies died, unlike the PICSO group. These findings require validation in larger, well-defined clinical trials. Whether the tested microRNAs are the sole signaling molecules or if other unexamined pathways contributed to clinical improvements remain to be determined.

On the other hand, if the claims of the embryonic recall hypothesis are valid, the primary effects of this intervention might be in treating heart failure. Compared with more complex procedures and concepts, PICSO could achieve widespread use, helping prevent worsening of heart failure and bridging the gap between traditional therapy and cardiac support devices. Recent results from a heart failure cohort have shown beneficial effects and provided insights into a possible mechanobiological mode of action and its connection to patient outcomes [45,46].

The miRNA pattern observed in heart failure survivors treated with PICSO should be interpreted in the context of disease stage, chronic myocardial stress, and complex remodeling signaling, in which developmental, inflammatory, and fibrotic programs coexist. While many mechanistic data linking these miRNAs to cardiomyocyte proliferation and angiogenesis originate from rodent or in vitro studies, the current findings nevertheless provide meaningful support for the “embryonic recall” concept: specifically, the upregulation of miR-19 family signaling and the miR-421–SOX4 axis is consistent with reactivation of transcriptional programs that are active during cardiac morphogenesis and early myocardial growth [60]. This was tested in rodents, and the respective miRNA 19 was injected into the myocardium. In failing adult hearts, where cardiomyocyte turnover is otherwise limited, such re-engagement of developmental pathways represents a biologically plausible regenerative response to altered mechanical loading and shear forces induced by PICSO. However, the simultaneous elevation in miRNAs associated with endothelial stress, such as miR-363-3p, which suppresses angiogenic proliferation, and reduces endothelial inflammatory response via inactivation of the *NOX4*-dependent p38 MAPK axis [61]. Together with reductions in fibrosis-related gene expression, pro-repair and survival-associated miRNAs, including miR-25-3p [62], miR-101-3p, which is downregulated in sera from patients with myocardial infarction [63], and miR-30d-5p, decreased in our study [46]. This may interfere with the cardioprotective effect in MI and could reduce the risk of developing ischemic cardiomyopathy [64,65]. This suggests that the observed response does not represent a complete reversion to an embryonic growth state, which is neither intended nor achievable. Instead, it reflects a hybrid remodeling phenotype, integrating elements of regenerative recall, as per our hypothesis [4,39]. With injury-adaptation signaling. Because adult myocardial ncRNA networks contain only partially annotated and context-dependent targets, and because most foundational mechanistic research is still based on rodent models, definitively distinguishing regenerative gain from compensatory adaptation will require prospective, controlled clinical studies using human myocardial tissue and tracking structural outcomes over time.

Unlike descriptive microRNA data in the translational research on cardioprotection and cardiac repair, there is a risk in choosing a singular pathway initiation, losing out on the complexity and matrix of interaction, falling short in the final intention to recover the heart, especially since the context of confounding signals seems to be pivoting in specific domains in regeneration [66].

Therefore, the findings support the idea that multiple signals may assist, including regenerative and intended developmental processes, cardioprotective signaling in the failing heart, and signals that interfere with the disease process in general. As shown in Figure 5, the goal of using PICSO is to halt clinical decline and reverse symptoms. Whether the observed clinical benefits can extend lifespan and eliminate the need for more invasive procedures should be the focus of future comprehensive evaluation trials.

The intended use of the PICSO intervention, as shown in Figure 3, extends from ACS to chronic heart failure, including all “therapeutic” components of PICSO such as redistributing flow to deprived areas, subsequent washout by oscillating venous flow, the plasma skimming effect at the entrance from large veins into venous microcirculation, and activation of regenerative pulses via deflection of cilia and stretching pericytes. It is an attractive venous catheter intervention because of the short application time required to induce signaling, inducing regenerative pathways. However, there is also a preventive option for patients selected for complex cardiac interventions.

## 5. Epilogue as a Prologue of the Future of PICSO

**Figure 6:**
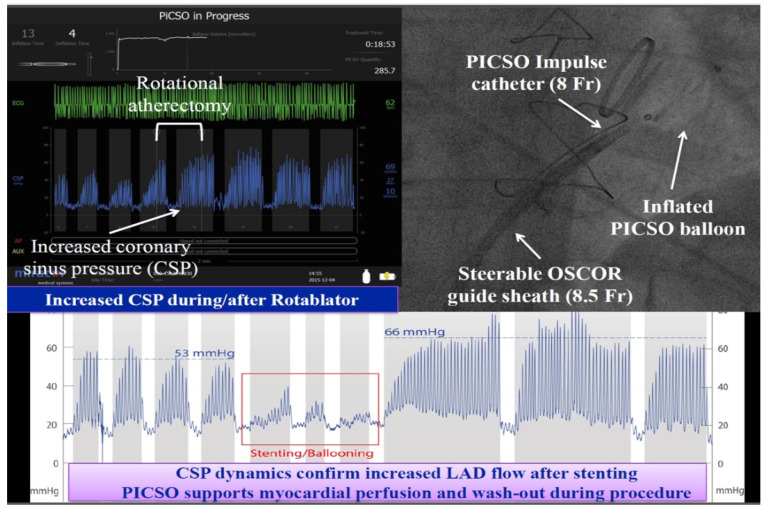
Patient during a CHIP procedure performed by Antonio Colombo, showing stable hemodynamics and increased LAD flow presented during a live case from San Raffaele during TCT 1016. Note the changes in coronary sinus pressure due to stenting and temporal occlusion of the coronary artery during ballooning. Coronary Sinus Pressure (CSP) increases because of the effective improvement in coronary perfusion with a higher plateau level.

Recent evidence-based clinical studies have been a substantial blow and a tidal wave against the current PICSO application [47]. There is, however, ample evidence that current therapy options extend from prevention of ischemia in complex interventions, to the use for myocardial infarction and treatment in heart failure, which needs thorough attention from the scientific community and interventional cardiology practice. To mention is the use of PICSO in severe shock and CHIP patients (Figure 6). Pappalardo used PICSO in a patient in shock and observed a critical increase in myocardial function, enabling the patient to be taken off cardiac ECMO support [67].

It is an attractive proposition to revive inborn mechanisms for building embryonic hearts, converting them into a regenerative impulse for adult failing hearts, as recently described by Wagh et al. [46]. From the perspective of the mechanistic action of clearing the ischemic microcirculation and its consequences in ischemic hearts, the additional benefit of this intervention may gain widespread acceptance, given the difficulties associated with cellular or other molecular therapies. Our understanding of the complexity of regenerative signaling involved is limited, and there may be even more pathways involved than have been tested. However, the intervention, in itself, as a revival of inborn mechanisms of canonical pathways, serves best in this context [13].

To effectively utilize PICSO in acute coronary syndromes, however, we must reconsider our expectations based on scientific evidence, focusing on earlier phases of acute ischemia. This entails searching for concepts and further developing the technology to enable earlier therapy initiation, even in the pre-hospital phase. This implies a current search for a safe, easy catheterization procedure that can be performed before or in parallel with reopening the coronary artery, a system that allows longer procedure times without blocking Cath-lab time, and the development of new algorithms and machine learning to optimize procedure duration and effectiveness.

Paradigm shifts in the perception of new reports of a molecular interaction, driven by intermittent pressure increases in cardiac veins, allow broadening the PICSO application to another unmet clinical need: inducing regenerative signaling in chronic heart failure. Early clinical observations are promising and should be followed. Although the mechanism of action must be corroborated, the early reports of PICSO in heart failure are promising, as the results open an unexpected treatment option for myocardial jeopardy and an avenue for in-depth evaluation, broadening the conundrum of reconnecting cardiac organogenesis with regeneration in heart failure. Since PICSO induces changes in the manifold expression of signaling molecules, a simple coronary sinus intervention may be a step forward in current molecular therapies that utilize specialized single signaling molecules, using RNA therapeutics to modulate regenerative pathways [68,69].

As with all other interventions in cardiology, rigorous monitoring of research branching points is necessary, and evidence and targets must be reassessed in response to paradigm shifts imposed or suggested by counterintuitive observations. Another sprint in a decade-long PICSO research marathon will modernize the technology and its expectations. Respecting previously unvalued knowledge will facilitate the subject of this therapy to the most rigorous clinical evaluation standards.

## Data Availability

No new data were created or analyzed in this study. Data sharing is not applicable.
